# Extra-Limites

**Published:** 1840-01-01

**Authors:** 


### EXTRA-LIMITES.

### AN INTRODUCTORY LECTURE,

DELIVERED TO THE

### ANATOMICAL CLASS

OF

### *SAINT GEORGE'S HOSPITAL*,

IN THE

THEATRE IN KINNERTON STREET,

*On the 1st of October*, 1839.

BY HENRY JAMES JOHNSON.*

YOU have heard, Gentlemen, from your able Lecturer on Surgery, the nature and the objects of your science. The Temple of Medicine has been built up before you, and you have seen that, in its various halls, as various lessons will be taught. Within this Theatre, the delight, or labour, of study has commenced, and its primary facts, its elementary characters will shortly be displayed before you.

* Perhaps an apology is necessary for printing so flimsy a sketch of the History of Anatomy as this. But some of the friends, and several of the pupils who honoured me with their presence at the delivery of the Lecture, having intimated a desire for its publication, I have complied with their flattering, and, I fear, their partial wish. My only hope is, that the same indulgence which overlooked its faults in the Theatre, may wait upon it in the press.

Censoriousness may possibly be disarmed by the admission, that the Author makes no pretension to the character of a historian. He merely aims at conveying a general idea of the progress of Anatomical Science, and exhibiting the principal phases of its growth. Those who are accustomed to listen to *introductory* lectures, or to *give* them, will confess that recondite speculations would be out of place, and will make most allowance for the defects which must be obvious in the present. My *readers* will be pleased to find that the Lecture has been compressed to its actual small proportions; my *hearers* will, perhaps, complain, that an equal kindness was not shewn to them.

Here you are to investigate the materials you are made of—the office of each organ—the attributes of every fibre—their subtle combinations—their admirable harmonies. Here you are to take the composite machine—to dissect—to analyse it—and to solve, or try to solve, the difficult physical, impossible moral problem—to know yourselves.

To a reflecting being, his own material constitution must, from the first, have been a subject of interest. But, in the earlier ages, superstition blindly, yet humanely, opposing the ferocious spirit of the times, fenced in with a false sanctity the bodies of the deceased. Denied the rites of sepulture, the unhappy ghost wandered around the spot where his dishonoured corse had lain, barred alike the privileges of the living and the dead.

At such an epoch, human dissections would draw down the vengeance of gods and men. Diseases were inflicted, and their cure must be effected by the Deity—his servant was the priest—the priest was the physician. Thirty-five centuries were already gone by, when Darius, the son of Hydaspes, received a simple luxation of the foot. He travelled to consult the skill of Egypt, renowned in art, at her acmé of refinement, the cradle of the sciences and gods of Greece. In the land of Apis, the deified discoverer of medicine, the dislocation of Darius was neither reduced nor understood.

Thirsting for a knowledge of the composition of animals, Pythagoras roamed over the known world. His pupil Empedocles treasured the doctrines of his master, and instructed his hearers in anatomy. He taught with confidence that " two parts of water, one of fire, and as much of earth," were the simple ingredients of the nerves, whose extremities, cooled and hardened by the air, formed, of course, the nails!

The first great revolution in medicine was at hand, for, after the lapse of a few olympiads, Hippocrates observed and wrote. The gods were the source of disease no more—the priests and the oracles ceased to prescribe—

> No voice, nor hideous hum
> Sounds thro' the arched roof, with strains deceiving;
> Apollo, from his shrine,
> Can no more divine,
> With hollow shriek, the steep of Delphos leaving.

Bacon was anticipated, for induction was applied to physic—and the genius of Hippocrates stamped it a science. You would naturally hope that the " Father of Medicine " was also an anatomist. He suspended, indeed, a skeleton of brass in the Temple of Delphi, a present to posterity and to the god. But a knowledge of the bones may be attained without dissection—it is certain that Hippocrates confounded the tendons and the nerves—and his physiological information may be guessed from the following sample:—The brain, he tells us, is a large sponge, whose appropriate office is to soak up the pituita, which distils, in chilly weather, from the nose!

Another century gave birth to a great captain, and to a greater man—to Alexander and to Aristotle. The soldier supplied the purse of the philosopher, and £200,000, and innumerable agents procured for him animals from every clime.

Of the Stagyrite, it might be said, in no sarcastic sense:—

Schœnobates, augur, medicus, magus, omnia novit.

His lessons have survived the admiration or neglect of ages, and are singularly fortified by modern zoology. But the power of his master failed, perhaps, to obtain for him the dissection of a single felon; for, Aristotle informs us that the nerves and the ligaments spring from the heart—that the brain is a mass of earth and water, without blood and without sensation—and that its only office is to balance and moderate the fire in the bosom!

Among the generals of Alexander, learning will ever honour Ptolemy Soter. We cannot feel surprise that the man who had listened to Aristotle, should found the Alexandrian Library. But the library was itself attached to the Musæum, and that was adorned by Herophilus. His name should be embalmed in the memory of anatomists, for he was the originator of human dissections. The popular hatred then, as since, pursued the benefactor of his species, and Herophilus was branded by terror and fanaticism with the guilt of dissecting six hundred criminals alive. The accusation, though launched by a Father of the Church, may be safely discredited by us, who remember that Medea, the inventor of warm baths, was reproached with boiling her patients in a cauldron. Among the merits of Herophilus, the discovery of the nerves was not the least. Modern description still appeals to him—and such was the enthusiasm of an earlier age, that Fallopius laid it down as a certain maxim—that, to contradict Herophilus on a question of anatomy was as impious as to contradict the Gospel!

They who have read the page of history but carelessly, may suppose that since dissections were begun, the progress of anatomy was rapid. But it shared the fate of philosophy, and drooped amid the mushroom crop of empirics and of sophists that sprang from the ruins of liberty and of the schools. The shadow of the Roman eagle overspread the earth, and science withered beneath its wing.

The severe investigations of Herophilus were shunned,—his memory was oppressed by ignorance and calumny—and religion prescribed laws to physiology once more. The Polytheism of Italy adored Ossipaga, the Goddess who regulated the growth of bones—and Carna, whose peculiar care was the bowels. Her most acceptable offerings were bean broth and bacon—the beau idéal of wholesomeness in ancient diet—the holiday fare of a Roman God!

The wheel of fortune had performed it's revolution—superstition again darkened it's zenith—anatomy was once more dishonored and denounced—and Asclepiades taught the compendious doctrine, that the structure, both of the body and the soul, consisted wholly of invisible particles and imperceptible pores!

But the reign of Trajan brought other blessings, as well as victory, and the conqueror of Dacia was the master of Galen. By a singular felicity, the restorer of anatomy flourished under three of the best of the Cæsars, and his labours were prosecuted amidst the repose which crowned the virtues of the Antonines.

Those labours were far from trivial. In the books of "Anatomical Administrations" which have been saved, may be discerned the germ of the modern discoveries in the functions of the nerves. I cannot resist one brief quotation, shewing, as it does, the influence of anatomical studies on a cultivated mind. The Pagan dissector breaks into this passionate address to Jove:—

"In writing these books, I compose a true and real hymn to that awful Being who made us all. And, in my opinion, true religion consists, not so much in costly sacrifices and fragrant perfumes offered on His altars, as in a thorough conviction in our own minds, and an endeavour to produce a similar conviction in the minds of others, of His unerring wisdom, His resistless power, and His all-pervading goodness."

However lax the discipline of Rome—however sunk the majesty of Jove—the infidelity of her philosophers (if she could be said to possess philosophy) was unable to vanquish the prejudices of paganism, fortified by the natural feelings of humanity. Even Galen, the great anatomist of antiquity, apologises for dissections of men, and points to accidents of time and war, as affording blameless opportunities. Where slavery prevailed in its most hideous form, where the Gladiators' schools, and the shambles of the amphitheatre exhibited their thousands—

"Butchered to make a Roman Holiday,"

the scalpel was seldom and stealthily used. Galen directs the student of anatomy to repair to Alexandria for the study of the skeleton—to visit the sepulchre, where bones may lie exposed—and to profit by the labours of the vultures on the bodies of executed malefactors.

I linger on the page of Galen, for scarcely had the urn received his ashes, when the volume of anatomical discovery was shut for thirteen centuries. The barbarians poured their swarms upon the empire—its mercenary bands turned their swords against each other—and the struggle between christianity and paganism ended in the growth of the papal power. Its pretensions were incompatible with reason and reason was proscribed. What the sophists had begun the priests concluded—the disputatious dogmatism of the schools was supplanted by the cruel dogmatism of the cloister—and a black and bloody superstition scowled upon the Christian West.

If the Church monopolised the little learning of the times, and preserved the embers of science from expiring, it was not for the love of knowledge, but for the liking of its profits. Men's intellects, darkened by ignorance, were dazzled and easily ruled by the priesthood, and it appropriated, as natural rights, the professions of physic and of law. How law was debased, let history tell—the degradation of physic was soon complete. Anatomy was almost held accursed, and the little that was taught or known was known and taught upon tradition. Medicine was a thing of herbs and charms—and surgery was consigned to the menials of the monastery, for the priest might draw no blood.

The Patriarchs of Constantinople envied, but could not rival the Bishops of Rome, and a faint image of liberty reigned in the schools of the East. Yet the subtle genius of the Greeks was averse from simple observation, and words, not things, were the subjects of their speculations. The sects were equal in number to the teachers,—the corrupted text of Galen the idola specus to which they bowed—and the only point on which all agreed, was *not* to study nature. Need I say that anatomy languished?

In the gloom of the seventh century, Mahomet raised the standard of the crescent, and, in less than thirty years the Koran and the small-pox prevailed from Mecca to Gibraltar. The sack of Alexandria by the Caliph Omar scattered its manuscript treasures, and the sons of Ishmael read, for the first time, the aphorisms of Hippocrates. The lapse of six hundred years and the virulence of a bishop maligned the Caliph's memory with the burning of the great library, because, "if it contained what the Koran did not, it was impious—and if it held only what the Koran did, it was unnecessary." Yet Mahomet, himself, was an empirical physician, and the liberal spirit of the Saracens refuted the malicious libel.

While Learning was banished from the West, she was harboured by those generous barbarians. The mind, however, of the Asiatics is more lively than solid, and has proved too imaginative to succeed in natural science. They admired, and imitated the speculative Greeks, and transcribed and glossed each ancient author. Rhazes and Avicenna, the Galens of their day, and long the worshipped luminaries of Europe contributed a little to medicine and to surgery—to anatomy next to nothing.

It was among the Saracens, that Alchemy arose. Ordinary phenomena appeared unworthy the notice of philosophy, and the marvellous alone was deemed a fit object of pursuit. But chemistry was rocked in the cradle of delusion, and this powerful agent of human improvement had its birth in fraud and folly. The key was found to the casket of nature, and Roger Bacon was the first to lift the lid. The efforts of bigotry and interest never after closed it, and the giant form of knowledge slowly issued, like the genius from the box of the Arab fisherman, till it stood before the world in all its majesty.

Great as have been the services of chemistry to medicine, it lent no immediate impulse to anatomy. Basil Valentine, however, and even Paracelsus, directed men's minds to the study of Nature, broke the bonds of authority and the dogmas of the schools, and laid open the new world of philosophical discovery, which adventurous observation might make its own.

The fifteenth century is conspicuous in the annals of our race. The art of printing gave immortality to knowledge, and the reformation struck the chains of superstition from the soul. The work which Roger Bacon had begun was carried on by Luther, and the reason, to which religious disputants appealed, was sharpened for the investigations of science. They were keenly followed, and the succeeding age saw bolder strides in anatomy and physiology than the fifty-six centuries that had gone before. Dissections were now notoriously practised—individual facts were observed and promulgated—and the agitation in the world of anatomy boded the coming revolution.

In the year 1531, a Spanish physician, by name Servetus, used these remarkable words:—

"The passage of the blood from the right to the left ventricle does not take place across the middle septum, as persons have generally imagined; it depends on a more singular structure. In the long winding of the lungs this subtle blood is agitated and prepared by the action of the viscus, and gains a yellow colour. From the pulmonary artery it passes into the pulmonary veins, where it becomes mingled with the air that has entered the lungs, and loses its fuliginous excrements. Lastly, it enters the left ventricle, which attracts it in its diastole."

The problem of the circulation was almost solved, reason alone might have worked it out, and we wonder how it escaped discovery. Yet seventy years separated Servetus from Harvey, and that long period was adorned by the talents and enriched by the observations of Vesalius, and Columbus, Eustachius, Varolius, and Ingrassias.

The story of Vesalius is so tragical as to claim a passing notice. A native of Brussels, he rose to be chief physician to Charles the Fifth, and to Philip the Second, his bigot son. His love of anatomy knew no bounds, and he was denounced by the priests and the Galenists as the dissector of a living person. The Inquisition was induced by Philip to spare his life, but condemned him to a pilgrimage to the Holy Land. The Senate of Venice invited his return to the professor's chair in Padua, but the anatomist was shipwrecked on the Isle of Zante, and perished miserably of cold and hunger. Vesalius, Roger Bacon, Galileo, paid, in their turns, the penalty of a genius that outran it's age, and sank under the iron arm of jealous superstition.

The plates of Vesalius are admirable for the period, and his descriptions generally accurate and elegant. A later anatomist (his name escapes me) on finding that some parts did not quite coincide with Vesalius' account of them, explained the contradiction by the ingenious remark, that "the structure of the body must certainly have changed since Vesalius wrote."

In 1563, Fabricius was appointed to that chair of Padua, which the unhappy Vesalius had been called to fill. His eloquence attracted students from all parts of Europe, and a young Englishman eagerly gazed, as he demonstrated the valves of the veins. The lesson was never forgotten—reflection suggested, experiment confirmed the brilliant conclusion—the secret of ages was finally disclosed—and the name of Harvey is imperishably linked with the immortal doctrine of the circulation of the blood.

Before the student of the present day has completed one course of anatomical lectures, he wonders at the dulness or perversity of intellect, which could fail to recognise so obvious a truth. He can scarcely be convinced that men of deep research and of powerful minds could seriously believe, in opposition to the evidence of sense, that the arteries contained only vital spirits—that the liver was the centre of the venous system—and that the blood went out in the veins by day and returned again by night. Yet such was their conviction, and it teaches us a truth which we never should lose sight of—how imperfect and how little trust-worthy the strongest faculties are.

The "circulation of the blood" was like the fulcrum that Archimedes sighed for. From it the world of physiology was stirred. The ancient hypotheses were shivered in the dust, and, in their place, uprose in natural order, the facts of modern observation.

The success of Harvey, like that of Columbus, tempted thousands to embark on the ocean of discovery, and the inductive method, the gift of Bacon, supplied them with the compass. The followers of the physiologist, as of the navigator, oppress by their numbers and their merits, and I am constrained to dwell upon those alone whose names are signalised by epochs.

Bologna and the Hague were adorned at the same time by Malpighi and by Ruysch. The doctrines of the Italian on the composition of the glands, though resting on the powers of the microscope, submitted to the superior fortune and wonderful injections of his rival. The latter was indebted to De Graaf and Swammerdam for the art which rendered him so famous, and which, since his time, has done such good service to anatomy. But much of his success was probably fallacious—other methods of investigation have corrected the defects and errors of injection—and the present day has made tardy atonement to the greater accuracy of Malpighi.

Injustice, even, would blush to pass Haller by. Of great capacity, of untiring industry, devoting himself with equal ardour and good fortune to classics and to botany, to poetry and to anatomy, Germany regards him as a modulator of her tongue, medicine reveres him as the first of physiologists. To his golden volumes, the preceptor and the pupil may still go back, and both will reap instruction from their philosophic spirit of experiment and reasoning.

A young man attracted the notice of Dessault by his lively talent and precocious knowledge. The surgeon of the Hôtel Dieu had discovered, his generosity fostered the brilliant, the imaginative Bichât. His was the soul that

"Frets the pigmy body to decay,"

for at the age of 31, he was lost to science and his country. Short as was his career, it endured long enough to revolutionise medicine. He conceived, or borrowed, the felicitous idea, that the different textures which compose the body have special laws of health and of disease, and general anatomy started almost perfect from his hands.

The structure and the functions of the human body had been hitherto almost exclusively studied. If Aristotle examined the composition of animals, he was encumbered by a false philosophy, and he had not the assistance of those great discoveries which I have feebly sketched. But the time was come for extending the inductive method to the anatomy of all creatures, and Cuvier and Hunter made themselves imperishable names.

On Hunter, it would be idle in me to dwell. His museum may be daily visited by all, and beneath his bust might fitly be inscribed the epitaph on Wren:—

"Si velis monumentum, circumspice."

His writings, alas, are an imperfect record of what he knew and of what he did; and when time has corroded, as, in spite of our solicitude, it must, the great conceptions realised in the museum, posterity will question the justice of our admiration. The learning he despised is already taking vengeance on his memory, and has wreathed her choicest laurels for his rival's brow.

The glowing page of Cuvier contrasts with the obscure and frigid style of Hunter. Its happy periods grace its noble images, and the inspirations of genius wear their most enchanting form. The minute details are only surpassed by the splendid generalizations, and both are tinctured by that sound philosophy, too frequently forgot by the lively temper of his country.

From the anatomy of animals to their physiology cannot be considered a step. The dissections of Cuvier and Hunter produced, ex necessitate, the researches of St. Hilaire, Blainville, Tiedemann, and Müller. Man is no longer, in the eye of the anatomist, that microcosm, ignorance and vanity supposed him. In structure and in functions he forms a step in the great natural ladder, and neither his position nor his office is intelligible by looking solely at himself.

His composite organism is made up of parts, some more, some less developed in him, than in beings lower in the scale, and problems of its nature and its properties find their analogical or direct solution in some other animals. Human physiology can no longer be studied, nor understood apart; but, merged in general or comparative physiology, it takes a wider range, asserts a bolder aim, and submits the whole organic world to its calculus.

A review of the progress of anatomy will shew that it has shared the fortunes of the other great departments of philosophy. Feeble amidst the ignorance of early times—repressed by a timid, and prostituted to its purposes by a base bigotry—lost in the darkness of the middle ages—it revived with learning's revival. Yet through all reverses, it contained within itself the elements of triumph, because the elements of truth, and sprang up most vigorous when destruction was most imminent. So certain it is that the battle of science, like that of freedom,

> once begun,

> Bequeathed, like it, from sire to son,

> Though baffled oft, is ever won.

I need scarcely advert to the spirit with which anatomy is to be studied. We must bring to it that ingenuous temper of mind, which natural science demands, and which Harvey, and Haller, and Cuvier, and Hunter brought. A temper of mind, whose generous aim is philosophic truth, which it seeks at any cost of consecrated authority, of cherished belief, and of those allurements of hypothesis, which are so seductive to us all.

To divest itself of every prejudice, whether of opinion or the senses, and to accept all conclusions, however startling, and however repugnant, which follow legitimately from data well-observed, is its constant object and its great prerogative. And those amongst you who pursue such noble ends by such worthy means will reap, I cannot doubt, the rich rewards of discovery and fame, for the volume of nature has been far from read, and many a bright page is yet to be scanned by her ripe and diligent student.

Your fatigue, gentlemen, is evidence how unworthy I have proved myself of the task I undertook. Langour would not have waited on the animated recital of an abler historian; but, lingering with him on the slow progress of science in former days, with him you would exult at its triumph in your own. The history of human knowledge is as instructive as delightful, and no liberal mind would willingly be ignorant of its strange and chequered course. The past, too, has its lessons for the future; and it is the god-like privilege of intellect to look towards both. The experience of a single life is an unit in the series of ages, and its errors are corrected, its truths confirmed by the undying experience of centuries.

### DR. FERGUSSON ON YELLOW FEVER.

*To the Editor of the Medico-Chirurgical Review.*

Sir,—My attention having been called to a late number of the Medical Gazette, wherein the infectious nature of tropical yellow fever is advocated in a well-written paper by Mr. William Fergusson, Surgeon of the Royal African Corps, I feel myself called upon, in vindication of the pledge I recorded when I last wrote upon the subject, in reply to Dr. Fraser of Demerara, now to examine the facts he adduces and the arguments he brings forward in their support. I feel it the more incumbent upon me to do this with as little delay as possible, because Mr. Fergusson is known to be an officer of great professional merit, who has much distinguished himself by useful services on the coast of Africa, and whose opinions, founded on much experience and submitted with diffidence and candour, cannot fail, if allowed to pass unquestioned, to have great weight not only in the medical world, but amongst all who are interested (and who is not?) in ascertaining the true nature of the disease.

Mr. Fergusson at once and unequivocally admits that it never was imported into Sierra Leona, the fountain spring and head-quarters of yellow fever—that there it always arises spontaneously, being truly endemic when it does come, and never introduced or sporadic; but that in the other settlements of the same station it never has been seen unless when imported from Sierra Leona, and that in the majority of them it never has been seen at all! This is a strange and a bold assertion, and before it can be admitted as a medical truth demands the strictest examination. Another of his propositions seems to be equally extraordinary—that the disease after being imported often requires an interval from 28 to 33 days before it can be made to shew itself amongst the inhabitants of its new field of operation. This is so contrary to all the known laws of incubation in febrile diseases, or indeed in any other diseases, with the exception of hydrophobia, that it cannot be received without the strictest scrutiny, and indeed is negatived by himself in the instances of Monsieur Imbert and others who were supposed to have imported it, at the shortest contact, into the Island of Goree; but let us discuss if we can the first of Mr. Fergusson's positions before we enter upon the second.

Yellow fever is acknowledged always to spring spontaneously at Sierra Leona; then why may it not equally so arise at Gambia, Goree and Ascension, when circumstances favour the development of the disease. Mr. Fergusson surely will not contend that Sierra Leona can possess an exclusive patent for the generation of yellow fever, and instead of this impossible incubation, would it not have been more reasonable to have allowed that the same causes which had produced the disease there, had, in the course of a month, been ripening into such accumulation as favoured its explosion at the other settlements of the same coast.

The last of these, from the fact of the Ban frigate touching there, and landing sick of yellow fever from Sierra Leona in the year 1823, soon after which the disease spread amongst the garrison, has ever presented the most difficult stumbling-block to the anti-contagionist; but from what we have seen of the leeward bases of hills in every part of the West Indies, or indeed of their bases whether to windward or leeward, we know that, what with drainage from the higher land and the difficulty or total absence of perflation, their lower levels are ever in a state to become the most fertile fields of accumulated malaria. To this Ascension, nearer to the equator by several degrees than Sierra Leona itself, can be no exception, and on the last visitation of yellow fever in that island (1838), the well-known causes of the worst tropical fevers, viz: the great collection and rapid drying of waters, had become accumulated in such abundance as to account most amply for any invasion of malarious fever, such as that of Sierra Leona itself, without having recourse to importation. The first case—that of the fever breaking out soon after the Ban landing the sick of it in the island, is a much stronger one: but one swallow does not make a summer—nor can a single instance ever be taken for proof. An interval of fifteen years elapsed between the arrival of the Ban and the second outbreak of the disease, and in that long period did no vessels from the head-quarter station ever touch there with fever on board, and did nothing of the kind, no sporadic instances, ever occur but on the above-mentioned occasions? I know not whether they have or not, but I know that, to constitute an important contagion, the disease must unerringly ensue upon the introduction—if it fails as often, or oftener than it hits, then must there reside in the locality itself a cause stronger than the arrival of infected ships, which independent of any such aids will, according to laws of its own, cause the disease to explode under particular circumstances of atmosphere and locality, as yet beyond our ken, and leave us in wonder and dismay.

I have never been in Western Africa, but I should think Gambia or any other settlement on the banks of one of its great pestiferous rivers must come under the same description as Sierra Leona; and Goree, when sufficiently peopled by Europeans to give scope to the disease, may be classed under the same head. This I shall now endeavour to prove, more especially in regard to the first of these, from the analogy of New Orleans in North America. Like the settlement of Gambia it is founded on the banks of a magnificent river amidst the alluvion of its mighty current, with the difference of its containing a crowded white population. For nine months in the year it may be called a healthy habitation, but when the heat of autumn ascends to that of the tropics, yellow fever appears with the regularity of the equinox and departs as regularly when it descends in the scale of the thermometer on the first approach of the winter rains. Being the greatest emporium of the Southern States, ships keep arriving from every part of the world at New Orleans, without regard to the state of its health, where they often lose their crews, but that is all—they depart with fresh men East, West, South, and North, and I never heard even the strongest contagionists assert that yellow fever was imported anywhere from New Orleans. The fact is notoriously otherwise. Her steam-packets meanwhile ascend the stream by hundreds, laden with fugitives from the malarious pestilence to the cooler localities above. When ill with the disease they vomit on the decks as they go along, but whether they live or die they neither infect their fellow passengers nor introduce the disease into the settlements of refuge. Now can Mr. Fergusson, when he reflects upon the fact of yellow fever being in the instance just quoted purely a disease of season and locality, really believe that it must be an imported plague when it appears on the coast of Africa always under the same circumstances of climate as the autumnal season of New Orleans? but I will give him another instance. At Vera Cruz, the nearest great town to New Orleans, but within the tropic (latitude 19°) it is ever at home to the European stranger provided he stays all night; but should he chance to move the smallest distance into the interior, yellow Jack, as our sailors call the disease, although prepared to fold him within his closest embrace had he caught him in the dark shades of the evening, always makes his bow at the city gates. Nothing can induce him to give convoy further, nor attend the stranger a foot of his way. Gambia and Goree, when we look at the map, may be called geographically, neighbouring stations to Sierra Leona, and when the climate is the same and the locality is either naturally, as on the alluvial banks of the river, or through the circumstances of season, made favourable, need we be surprised that when it prevails in the last, where, in Mr. Fergusson's own words it is "ever the undoubted product of the colony itself," it should also pervade the latter without the intervention of ships carrying the disease? Many, indeed I may say all, colonists will strive to remove the reproach of pestilence from their own shores. They will greedily imbibe the grossest delusions—swallow and propagate the idlest tales of importation, and shut their eyes to the most obvious facts of native origin, for as long as they can make the favourite belief subservient to the character and interests of the colony, consequently to their own. At Sierra Leona when the pestilence was springing up rife from under their feet, and before their eyes, Mr. Fergusson tells us they strove hard to discover the ship that brought it to them—but it would not do. Gambia, however, thinly peopled by Europeans, more remote, and less under observation, had more of its own way and took the consolation of discovering the importers in the persons of Mons. Imbert and his friend. The flattering unction could not be resisted, and I regret to perceive it has pervaded even the enquiring mind of Mr. Fergusson. That all this at Gambia must have been a delusion, and that yellow fever in every situation must at all times have been a disease of high temperature and locality alone, let us now seek for what may be called European proofs nearer home, and better suited to the strictest investigation; and here I will take my demonstrations from the evidence of Sir William Pym himself, at Gibraltar, by far the most consistent and ablest advocate on the contagion side of the question. In his celebrated work on the Bulam fever, which has ever since so mischievously influenced all the health departments, indeed I may say the government of the country, at p. 51, he thus tells us how he arrested the progress of a yellow fever :—

"I directed a sufficient number of tents to be pitched outside the gates of the garrison, on the neutral ground, with a proportion of bedding, &c. and in the middle of the night without any previous information being communicated to the inhabitants, a strong guard with a sufficient number of carts proceeded to the infected district and conveyed the different individuals of the infected families *sick and well, with their baggage,* to the encampment which was established as a lazaret and kept in quarantine. I placed the sick in separate tents and appointed persons to attend them who had had the disease at a former period. A cordon of troops was also established round the infected part of the south district, which was kept in quarantine for fourteen days; proper persons were also appointed to superintend the purification of the houses, furniture, &c. and to report the slightest appearance of disease among any of the inhabitants—who were also paraded and inspected daily by one of the hospital staff."

Now can any thing be more evident, from this quotation, than that the fever was one strictly of locality in the town of Gibraltar, and that even with the aids of the diseased bodies, and all their baggage, it could not be implanted at the shortest distance beyond the gates of the fortress; for had the disease been small-pox or any other true contagion, it might, in the fomites of the sick, have been carried round the world.

The measure, whatever may have been the understanding and the motive, was one of great practical wisdom, and ought to have afforded an excellent lesson in all the subsequent invasions of the malady, for a move even to the shortest distance at once stopped the disease, and furnished the most unanswerable reply to the doctrine it was intended to establish. Wherever pestilence, whether malarious or not, takes up its abode, it is a maxim of old standing to depart as soon from the place, go as far and stay as long as convenience will permit; but when large bodies of men, such as a garrison, or encampment of troops cannot so migrate, the smallest change of locality, as in the instance before us at Gibraltar, will often suffice to stop the disease; and there ought always to be a supply of tents in the garrison adequate to the occasion, whenever the disease arises again, as unquestionably it will, whenever the temperature of the season and the state of the rock favour its explosion. It has been triumphantly asked, why, if Gibraltar hill in its different aspects of becalmed locality and leeward base be so favourable to the production of yellow fever, Cadiz, perfectly flat on a dry isthmus, should have been visited by the disease fully as often? and the reply is obvious. All walled towns become depôts of accumulated malaria in the countries where that element of disease exists. Their walls and defences impede ventilation, and render perflation all but impossible. The poison collects at the base of the fortifications, and lurks in the angles and ditches of fortified lines. Hence Seringapatam in the East, not very unlike Gibraltar in its form, has ever proved a most dangerous quarter to Europeans. Hence Barcelona has more than once been visited by true yellow fever,* and hence have Fort Bourbon and Fort Edward in Martinique, and Fort Fleur d' Epé and Fort Matilda in Guadaloupe, ever proved most deadly to their garrisons. I may be told that when all the circumstances just related have been found in the fullest operation, not only for a season, but for courses of seasons, no yellow fever has been produced, and but for its introduction by ships it never would have occurred. To the first I assent, but the second is an invention or a delusion. Who can say, in our more fortunate land, why malignant, epidemic erysipelas, or fatal puerperal fever, should pervade whole districts in one season, and in another of no perceptible difference, or even more favorable aspect, neither the one nor the other can be called into existence: and are we, when in malarious countries, because we cannot tell why yellow fever should absent itself for a long or a short period, justified in asserting, when it comes, that it must have been introduced from abroad; proclaiming, consequently, the advent of a new plague, and denouncing the sick as being unapproachable and dangerous, for once make the people believe in the reality of imported pestilence, and we shall call upon them in vain for succour and assistance to objects breathing forth contagion and death to all who approach them.

With this question of tropical contagious fever I have often grappled, and I shall now try to grapple again. True contagion is no respecter of persons but the negro race, indeed I may say the whole coloured population, the most numerous by at least ten to one of the inhabitants of our West India Colonies, never take the disease; and the native Creole whites and creolised inhabitants, relaxed by the climate and enervated in their constitutions, therefore less fitted

---

* I will here venture to prophesy that, if ever the heat should amount to that of the tropics, and be continued for a sufficient length of time, St. Sebastian, from its form, will become the seat of yellow fever. Its beautiful tide river, through which our troops at low water waded to storm the breach, has hitherto to a certain extent been a safeguard.

to resist any new infection, are infinitely less liable to be attacked than newly-arrived Europeans. I have read of negroes taking yellow fever when returning to the colonies after a residence in cold countries, but though I have twice in the course of my life sojourned for a course of years in the West Indies, I never saw it, nor met with any one who had actually seen it; and is the negro infection-proof? Have the true contagions of small-pox, measles, scarlatina, &c. no power over him? or typhus fever when in this country, or plague when in the Levant? and can he resist the infections of syphilis and scabies any where? These questions would be ridiculous, if seriously propounded, for it is notorious that whole plantations have often been ravaged whenever small-pox and the true contagions have been introduced. Why then does he resist the supposed contagion of yellow fever?—simply because it is not a contagious but a malarious disease; and against all malaria it is well known that he is fever-proof: but let us examine this question if we can a little more deeply. The contagion of fever does not readily take root under tropical heat—our typhus fever cannot easily be carried to the West Indies, or if it should in foul transport ships, perchance, be landed there, it is immediately dissipated by the heat of the climate. The high temperature is most prolific of fevers, but not of contagious ones. The late Dr. Henry, of Manchester, proved by positive experiment that of all disinfectants heat was the speediest and most infallible. He exposed the gross tangible contagious matter of small-pox, cow-pox, and scarlet fever to a heat of 140° of Fahrenheit, and found that they were thereby deprived of all infectious power; and in the case of typhus fever not being transportable to the tropics, we had, during the last war, more particularly in the ill-fated expedition of Admiral Christian, in the year 1795, proofs innumerable that the gaseous, ærial contagions were dissipated under a heat of between 80° and 90° of the same scale. Having been there, I speak from actual observation. We know, moreover, that the plague of the Levant disappears as soon as the high Summer heat in Egypt sets in,* and that it never yet has been carried beyond the tropic into the equatorial regions. Now should not these facts (for they are facts) weigh with Mr. Fergusson, before he propagates the dangerous belief of yellow fever, with the exception of Sierra Leona, (and why that exception) ever being an imported contagious pestilence, as if contagion could be the product of the disinfecting principle itself! I have never enjoyed the honour of his acquaintance, but I duly appreciate the usefulness of his life, and, in the hope that he may yet have a long career to run, I entreat he will give them the consideration they are entitled to, and then I am sure he will no longer lend himself to a delusion, which, wherever it can be made to prevail, must be attended with consequences at which humanity shudders. Let him reflect that the word contagion, always the word of fear, if pronounced by him, who has had so much experience, may prove the word of doom; and that it will be far more honourable to acknowledge that he may have been mistaken, than to persist in an error which, if received as a truth, must prove fatal not only to many of the present generation of Europeans inhabiting the tropics, but to thousands yet unborn.

I abstain from repeating the arguments I brought forward when I last wrote in the Medical Gazette, in reply to Dr. Fraser. They are dated February 24th and April 24th, 1838, and also United Service Journal for April, 1838, and I beg Mr. Fergusson will do me the honour to read them, and then, when he returns to the coast of Africa, the field of his usefulness and research, he will I hope, on the next invasion of yellow fever, take for his guide the emphatic words of my friend Dr. Gilkerest, of Gibraltar—PLACES NOT PERSONS—for they comprehend the whole history of the disease, in its source, nature, and

---

* Vide Assalini.

progress. The cruel quarantine will then no longer be made to vex the coast, and the courage of the inhabitants, when they come to know what the disease actually is, be assured. They will then, instead of denouncing one another, seek, in as far as they can, the only rational means of safety in a change of abode, but if the panic of contagion be kept up, then adieu to all the feelings of common humanity, no matter how humane and christianised they may originally have been, for self-preservation in all ordinary minds is ever the first law of nature. The sick will be deserted, thereby aggravating the malady a hundred fold, at the risk, too, of generating a temporary contagion of neglect and accumulation; for such is the nature of all febrile contagion (I speak not of the exanthemata) even within the torrid zone. All this will be in Mr. Fergusson's power whether for good or for evil, and I cannot doubt that he will choose the better part, for the superstition of contagion in tropical fever, the remnant of an age gone by, although still taught in our schools, is fast passing away, and I trust he will no longer be influenced by it. The doctrine is eminently antisocial, and if the facts and arguments I have adduced in the present and other writings to which I have referred him, are not to be refuted, I am sure he will acknowledge it to be equally unphilosophic. It is surely full time that the fumigators—the burners of bedding—as if the heat of boiling water at 212° would not suffice—and all the ill-omened hosts of quarantine, should be referred to the beautiful demonstrations of Dr. Henry, which must soon lay them to rest as effectually as the ghosts which frightened our forefathers were said to have been laid in the Red Sea. These experiments are pregnant with the most important results to the best interests of mankind in every part of the world, and will embalm his memory for as long as science and humanity are appreciated amongst the friends of the human race.

Having now, I believe, replied sufficiently to the letter of Surgeon Fergusson, I shall proceed to some general considerations of the nature of tropical fever.

In the examination of this question it seems hard that we, who have lived for years under the direst scourge of the disease, and sustained its attacks in our own persons under the most appalling forms, should always after a time be set aside as if we had never spoken nor written on the subject: and that the schools teaching contagion without ever having seen yellow fever, and the home authorities should, like the resuscitations of animal magnetism, again reassume their sway—setting at nought the evidence of experience and the accumulated testimonies, all but unanimous, of the fleets and armies who were so severely victimized by it in the course of last war. In the year 1795 England put forth her strength, and a magnificent armament that might have turned the tide of war on the Continent was arrayed under Sir Ralph Abercrombie for the recovery of sugar islands and the conquest of St. Domingo. To the latter I was attached, and its fate was the same as has ever attended, and ever will attend, a European army proceeding to the West Indies—the troops died by thousands, and if ever yellow fever and black vomit reigned supreme, it was then and there: but did that make us contagionists? On the contrary, it unmade us I may say to a man, and cured us for ever of the delusion that had been imposed by all the schools and authorities at home.

Dr. Bancroft has given the history of contagion and its short-lived reign in the windward colonies—Doctors Jackson and M'Lean that of St. Domingo; and although nearly half a century has elapsed, the inspectors general, Doctors Theodore Gordon, Sen., Borland, and Warren, who served with me there, are yet alive to prove the non-existence, the impossibility of contagion, and this I can confidently say was the thorough conviction of all, whether medical, military, or civilian, who had been obliged to remain a year or longer in the country. What we saw every day of our lives was *la fièvre Européenne* of the French—(la fièvre des blancs)—that most emphatic comprehensive phrase which in one word truly defines and characterises the disease. All history too confirms it:

turn to our annals, and it will there be seen that the expeditions to Carthagena, the Havanna, St. Domingo, and indeed every other to the West Indies, whatever might be the event of war, had but one termination—the burial of the troops from yellow fever. Hereafter Great Britain, unless she means the same tale of woe to be rehearsed for ever, must condescend to conquer, and more especially to preserve her conquests, with the black man's instead of the white man's arm —so much for the Army.

In the Navy it may fairly be supposed that the medical staff shut up in the same ship with the sick could scarcely fail to know whether the distemper was contagious or not; and although not so unanimous on the subject as an experienced army medical staff, are yet by a great majority anti-contagionist. The dissidents are to be accounted for from the comparative rarity of yellow fever on board-ship, (for it is a land-disease,) where they only see it as it is brought from the shore, or as it comes in bursts or explosions arising from the state of the hold or cargo or greenness of the timbers; and if it be the first of the kind witnessed, it may be difficult for any one, whether medical or not, to discriminate between the current of epidemic and contagious disease. I have already, in the Medical Gazette of last year and other publications, given the history of imported yellow fever at Barbadoes in the instances of the Regalia transport and the Childers sloop of war, both under my own particular care and direction, and shewed the impossibility of their being so much as suspected, after the freest communication of having communicated any contagion; and to illustrate these points still further, I shall here relate two experiences of what I may call marine yellow fever—the first occurring to myself, the second to a professional gentleman* now in Windsor.

In the year 1816, while yellow fever was raging at Barbadoes, it was my lot, I suppose, from being an old seasoned subject, to be taken with very violent but irregular intermittent fever. Admiral Harvey invited me to go on a cruise with him amongst the islands in the flag-ship, and soon after leaving Carlisle Bay, yellow fever broke out amongst the crew. There were, if I recollect right, seven very bad cases; but Mr. Neale the surgeon, a sensible and excellent officer, at once negatived the smallest idea of contagion. The assistant-surgeon, Mr. Byrne,* brought up his cot from the cockpit and slung it in the sick-bay, in order that at all hours he might be near his patients. Their comrades were allowed the freest access, and the officers of the ship were encouraged to visit and shew them every attention. The consequences were the happiest; for although the cruize lasted several weeks, we did not carry back a single case of the disease to Barbadoes.

The other experience to which I allude took place at Kingston, or rather Port Royal, Jamaica, where my friend was taking his passage along with a detachment of artillery and some officers of a West India regiment to Belize, in the Bay of Honduras, in the Recovery brig, commanded by Captain Hamlin. The yellow fever had been raging in Jamaica in the same manner as at Barbadoes, where the Recovery had buried three of her crew; and on the second day of the voyage, the 1st mate sickened and speedily died, under the most appalling form of the disease. The crew were panic-struck, and, reporting themselves sick, lay down upon the decks, and the captain, believing himself doomed, took to his bed. My friend luckily, having when at sea amused himself in taking observations, assumed the direction of the ship's course, and, favoured by the

---

* Mr. H. Moore, Dentist.

* I write from memory, and trust that I do not mistake his name, for his conduct was beautiful. I know no better term to express this tribute of my respect, and if in life I hope he will accept it. I believe he afterwards sailed with Capt. Basil Hall, as surgeon of the frigate he commanded.

winds, she reached Belize, where, as soon as the pilot had made his report, her arrival was looked upon as that of the death-ship, and she would either have been kept in the deadening quarantine or forced to sea again, had not the quarantine master, Staff-surgeon Thornton, fortunately been a man of observation and knowledge. He visited the sick, and declared that there was not a case of yellow fever nor any thing like it amongst them—they were merely panic-struck, and as soon as they received the hospitality and succour of the port, which they did without precaution or reserve, they were speedily all well again. Now in the first case, when the epidemic presence was upon us, had the medical men on board lent themselves to the delusion and cried contagion, we might, I verily believe, have unmanned the British flag, and converted the admiral's ship *(silvæ filia nobilis)* into a pest-hulk; and in the second place, there can be little doubt that had the ship been kept out at sea, or laid up in quarantine with the plague-mark upon her, disease would have done its work amongst the terrified people, and we then should have had a new edition of contagion, as in the celebrated case of the Hankey, arising from the deceased mate of the ship. One instance can never be taken for proof, nor do I wish what I am going to say to be held for more than it is worth, but on this cruise, and on other occasions, I saw hospital patients with ordinary diseases exchange beds with the sick dying of yellow fever, without any consequences resulting. I have even in the course of my life been obliged, through necessity and fatigue, to submit to something of the same kind myself, and I declare that, but for the personal impropriety, I should not feel the smallest hesitation to do it again, or to sanction its being done by others. In the St. Domingo army the disease, from some unexplained peculiarity of the time, rarely attacked the women attending upon and sleeping with their dying husbands, and the number of the latter some of these females had expended, for short as their wedlock had been, their widowhood was far shorter, became a constant source of jocularity and remark.*

That there are anomalies and difficulties in regard to the question of yellow fever—its sudden bursts and invasions—its disappearances too when within the Tropics, without in so far as we can see, any adequate reason, there being no such bursts within our recollection in Bengal, nor on the shores and islands of the East Indian seas, I freely acknowledge; but it has not been altogether unknown there, for one of its earliest designations was the *maladie de Siam*. In the East they have never dreamed of contagion in any tropical febrile disease, and that it ever should have become our creed in regard to the West must have been occasioned by European troops being landed there in large masses, and despatched on service throughout the colonies in crowded ships, thereby generating, it may be, although I can scarcely believe it under such a heat, for the passing day, a contagious atmosphere of accumulation. I will even go so far as to admit the possibility of this occurring in densely populated towns and garrisons such as Gibraltar, but the difference of a contagious atmosphere and a contagious individual is this. In the first almost all will be liable to fall under its influence while they remain in the place; but in the latter case, take the worst of the sick that can be selected out of the contaminated locality, and you may approach him as safely and receive as little contagion from him as you would from a marble statue or a graven image. True contagion infects at the shortest interview, and through the instrumentality of the smallest particle even of an invisible gas, in all situations and places: but factitious temporary contagion can only acquire power through the medium of a crowd generating ac-

* The case was widely different at Barbadoes in the year 1816, when the women, more especially those of the newly-arrived Queen's Regiment, died fully as fast or faster than the men.

cumulation. In the East the approach to its coasts was interdicted to all colonists under the monopoly of the old East India Company—crowds of Europeans never went and never resided there—nor do they even now; but when the suburbs of towns come to be filled with them, as at Gibraltar, it will then be seen, for it is eminently the disease of high temperature, whether the maladie de Siam may not again arise even in Calcutta.

These questions become deeply interesting when we reflect upon the present condition of by far our richest colony of the West (Demarara), where one of our finest regiments is reported to be under a course of speedy extermination from yellow fever—while I write even now the work may be accomplished. When it ravaged that colony, three years ago, Doctor Fraser, the quarantine master, true to his calling, declared that it was imported from Kerrynane,* or some such name, that it was fearfully contagious, and that all were Atheists who did not believe it.† To have imported the disease into such a place would indeed have been a work of supererogation, for a deeper—a mightier swamp I never contemplated. It may be called the deepest in the world, and the terms and periods of healthiness, instead of unhealthiness, at Demarara constitute the wonder: and here I feel myself called upon to speak of malaria as emanating from marshes and of vegetable putrefaction as constituting its element. When at the head of the medical department in the West Indies during the years 1815, 16, and 17, I made it my business, and I considered it my duty, to explore in as far as was practicable every swamp in the inhabited quarters of the windward and leeward colonies, and the result perfectly corresponded with that stated in the Government medico-statistical report—that even the most declared open swamps did not at all times produce disease amongst the inhabitants dwelling in them or near them. My experience led to the conclusion that for as long as they were filled with water they were generally harmless, but whenever they, or part of them, came to a dried or an advanced drying state, they were as generally pestiferous. In regard to vegetable putrefaction, as evinced by the smell, it absolutely meant, or at least produced nothing, no matter how strong the fœtor, and as I have stated in former publications, I have experienced it so strong as to deprive me of sleep. The most declared swamps in the fullest process of vegetable growth and decay were under the above circumstances innoxious, but so were not the deep ravines—the dried watercourses of hills, or the leeward bases of mountains, which perflation could not reach—these I may say were uniformly pernicious to all the white race of mankind. Swamps do not always produce malaria in tropical climates, but these never fail, and I believe never will fail, either there or in America or Europe, for as long as the steady atmospheric heat without rain amounts to between 80° and 90° of Fahrenheit. I am not pretending to say what malaria is, nor whether it be the same element as marsh miasma or not—the marsh so abounding in one may, when approaching to dryness, emit both—the ravine, if there be two elements, only one: the marsh, when filled with water, may prove as habitable as any other land—the ravine, more especially its bouchure, never: the marsh is notorious for producing ague, and the lesser grades of what has been called malarious fever—the ravine only the higher; it would even appear to be incapable of giving rise to the former, and when the time for invasion arrives, makes itself felt in bursts and explosions, often without previous warning, of such fevers as have destroyed our armies and devastated our West India colonies: the cleanest lands will not at these times save the inhabitants; for the dry rocky watercourses—the purest

---

* I had heard of Derrynane, but where Kerrynane is I have never been able to discover.

† This is much such an argument as was used by Lord Peter in Swift's Tale of a Tub.

sands, after they have been saturated with water, and the hard mountain basin, where vegetation never grew, become its constant abode, *provided always that they are to leeward and cannot be ventilated.* Vegetable putrefaction! as if our dung-heaps and pig-styes in every part of the world did not teach us better. Where was it in the arid plains of Spain, long denuded of all vegetation, when our armies were paralysed by autumnal fevers?—where is it even now in the dried pestiferous Campagna di Roma?—and why did it sleep when in healthy, (comparatively speaking,) because rainy seasons? I visited and reported upon the putrid marshes of Trinidad, Berbice, Guadaloupe, Tobago, and St. Lucie. Malaria is in fact the production more or less of the earth's surface—never of the ocean's—in every hot season or climate. It is certainly absorbed by water and dissipated by the winds, but when these agencies fail, it accumulates into force, and spreads disease and death around. We do not yet understand it, and never will understand it until we can clear our heads of the long-cherished superstition (for it deserves no other name) of vegetable putrefaction being the sole and only source of malaria. The leaven of the false creed will otherwise continue to prejudice our minds, and when disease, for which in the absence of marshes we can discover no cause, makes its invasion, constrain us to seek refuge from the trouble of thinking in the doctrines of contagion, and the monstrous safeguards of quarantine—a quarantine, in fact, against the heat of the season calling into existence a morbific element of the natural world. I am not pretending to lay down the law to others—I am only telling what I myself have experienced; and if the testimony of an eye-witness can be of any avail, the authorities, when Gibraltar again falls under the dominion of pestilence, as fall it will whenever the drought and calms are long continued, the garrison numerous and the heat great, may, if they choose to enquire, find the source of the disease in its hollows and fortifications and leeward base, without seeking for its importation from beyond the Atlantic: the fabled poison of the far-famed upas tree is but a type of malarious locality. I believe it grows in the unventilated mountain-hollows of dry rocky deserts, where the malaria is so deadly, but the tree itself, though exuding, when wounded, a narcotic poison, under the form of a white milky juice, is as safe to approach and to handle as ever a yellow-fever patient was in St. Domingo, and I believe I may add with confidence, in Gibraltar: and is this all declamation, and have we no remedy? It stares us in the face. Let yellow fever for ever be expunged from the list of importable and transportable contagions; the whole world of its range, as we see at this moment at New Orleans, Mobile, Augusta, &c. (and can a ship, with incomprehensible punctuality, have gone to all these places just at this season, as if for the express purpose of dropping yellow fever) is every year proclaiming the impossibility—dismiss, in as far as that disease is concerned, the ministers of quarantine, for they are paid and appointed to deceive—wherever it prevails seek the only possible safety in removing from the locality, so often a very limited one—and when that cannot be done, firmly await its sure extinction in the changes of the season, and eschew for ever the unchristian, I may in its consequences call it the atrocious doctrine, of excommunicating the sick, for in that way you may, through neglect and accumulation, generate a true contagious pestilence, which atmospheric heat without ventilation will neither dissipate, nor cold extinguish. Do this—I address myself to our rulers—and the whole race of importers and expurgators and fumigators* will no

* God forbid that I should be supposed to utter a word in derogation of discipline and cleanliness, but of all absurdities fumigation against we know not what (for who can tell the nature of those vapours that convey diseases) seems to me the greatest. In the application of caloric we possess a certain and immediate disinfectant, and in the admission of light, air, and water, we more be seen, for men's minds, redeemed from panic and prejudice, will then be turned to the lessons of true philosophy, and the British name be rescued from the reproach of having so long cherished a delusion so discreditable to the national character, and injurious to our best commercial interests.

I am now past the age and the ability of active service, and must leave the swamps and ravines of the Tropics to be explored hereafter by abler men; but I can still stand sentry at the gates of the printing office, and when illicit matter, pregnant with delusion upon the public mind, is passing through its engines, refuse it currency until subjected to examination, and bearing my protest: and with this protest I shall conclude, although I believe that, even at the risk of being deemed garrulous, I shall trespass on your pages with another letter, for even while I now write fresh matter, the recollections of times long ago keep rising to my mind, which (from self-partiality it may be) I cannot help thinking may prove further illustrative of tropical fever—its sources—its phenomena—its prevention and treatment.

W. FERGUSSON.

*Windsor, October 10th,* 1839.

---

### Dr. STRANGE ON THE CLIMATE OF NAPLES.

*Sorrento, July 8th,* 1839.

Dear Doctor Evanson,

I transmit to you a complete copy of the Meteorological Tables, which I showed you at Naples; and of which you expressed a desire to have a copy. You will find enclosed—1st, A copy of the meteorological observations made daily by me at Naples and Sorrento, during the year 1838.*—2nd. An abstract of these meteorological observations—and 3rd. A table shewing the mean of the hygrometer and thermometer, &c. &c. With such general remarks on the climate, and on the mode of making these observations, as I thought would prove useful.

Your's, very truly,

R. STRANGE.

---

have others equally sure but of slower operation, and indefinite with respect to time—why then resort to fumigation, even if it were proved to be a disinfectant (which it is not), unless to mystify the people. The trick has had its day, and is becoming stale—it has been too long practised, as I know from experience, for on my last return from the West Indies, in the year 1817, I was subjected to personal fumigation on three succeeding days at the quarantine station in the Isle of Wight, and in confoundedly cold weather too, lest I should introduce the yellow fever into England!!!

* These Tables are in the possession of the Editors; but are too voluminous to print in this Journal. The abstract is quite sufficient.—*Eds.*

*ABSTRACT of the METEOROLOGICAL OBSERVATIONS*

| MONTHS. | PREVAILING WINDS. Number of Days from each Quarter. | | | | | | | | RAIN GUAGE. | | | |
|---|---|---|---|---|---|---|---|---|---|---|---|---|
| | South | South-west | West | North-west | North | North-east | East | South-east | Number of Days on which Rain fell. | Quantity of Rain in French lines & decimals | Mean of the differences between the dry & wet Thermometers | Mean absolute dryness according to Dr. Mason's calculation |
| JANUARY.... | 8 | 6 | 2 | 4 | 2 | 2 | 2 | 2 | 22 | 50·19 | 4·73 | 11·01 |
| FEBRUARY... | 2 | 10 | 4 | 4 | 1 | 4 | 2 | 1 | 21 | 115·67 | 3·70 | 8·61 |
| MARCH ..... | 3 | 7 | 3 | .. | 4 | 5 | 4 | 5 | 21 | 41·99 | 3·84 | 8·94 |
| APRIL ...... | 5 | 11 | 2 | 3 | 1 | 2 | 1 | 5 | 10 | 41·27 | 5·70 | 13·28 |
| MAY........ | 6 | 13 | 1 | 4 | 4 | .. | 1 | 2 | 6 | 10·98 | 5·80 | 13·50 |
| JUNE ....... | 5 | 12 | 6 | 1 | .. | 2 | .. | 4 | 4 | 6·04 | 4·93 | 11·52 |
| JULY ....... | 4 | 11 | 9 | 2 | .. | 1 | 2 | 2 | 2 | 1·31 | 5·70 | 13·27 |
| AUGUST ..... | 8 | 10 | 8 | 3 | .. | 1 | .. | 1 | 4 | 16·35 | 6·77 | 15·77 |
| SEPTEMBER.. | 9 | 5 | 9 | 2 | 1 | .. | 1 | 3 | 4 | 11·37 | 4·65 | 10·81 |
| OCTOBER.... | 3 | 5 | 5 | 2 | .. | 11 | .. | 5 | 10 | 36·77 | 4·83 | 11·22 |
| NOVEMBER .. | 4 | 7 | .. | 4 | 5 | 5 | .. | 5 | 14 | 90·59 | 4·60 | 10·72 |
| DECEMBER .. | .. | 3 | .. | .. | 7 | 10 | 3 | 8 | 10 | 82·68 | 4·26 | 9·84 |

*made at NAPLES and SORRENTO during the YEAR* 1838.

| HYGROMETER. | | | | THERMOMETER. | | | | | | | |
|---|---|---|---|---|---|---|---|---|---|---|---|
| Greatest absolute dryness observed during the Month | Mean Dew Point | Highest Dew Point observed as the mean of a day | Lowest Dew Point observed—mean of a day | Maximum of the month | Minimum of the month | Mean maximum | Mean minimum | Mean Temperature | Mean Diurnal Variation | Greatest Diurnal Variation | Least Diurnal Variation |
| 28th, 21·00 | 41·43 | 50·34 | 30·17 | 62·00 | 38·00 | 57·30 | 47·60 | 52·45 | 9·70 | 18·50 | 2 |
| 19th, 22·16 | 43·21 | 51·67 | 32·50 | 64·00 | 41·00 | 56·28 | 47·36 | 51·82 | 9·00 | 15·00 | 3 |
| 31st, 26·50 | 45·26 | 53·00 | 31·00 | 65·00 | 41·00 | 60·10 | 48·31 | 54·20 | 11·80 | 21·00 | 5 |
| 24th, 28·00 | 42·17 | 50·17 | 31·67 | 70·00 | 43·00 | 62·30 | 48·60 | 55·45 | 13·63 | 20·00 | 6 |
| 2d, 37·33 | 48·72 | 61·00 | 40·00 | 83·00 | 51·00 | 69·00 | 55·51 | 62·22 | 13·50 | 24·00 | 6 |
| 9th, 31·50 | 55·68 | 64·34 | 36·17 | 81·00 | 55·00 | 73·44 | 60·96 | 67·20 | 11·25 | 15·00 | 6 |
| 15th, 30·33 | 57·81 | 64·84 | 45·00 | 82·00 | 59·00 | 77·00 | 65·16 | 71·08 | 11·84 | 15·00 | 8 |
| 12,16,18, 31·50 | 54·84 | 65·67 | 45·17 | 80·00 | 58·00 | 76·38 | 64·84 | 70·61 | 11·55 | 15·00 | 8 |
| 8th, 9th, 28·00 | 56·55 | 61·84 | 49·00 | 81·00 | 56·00 | 73·30 | 61·43 | 67·36 | 11·86 | 16·00 | 8 |
| 6th, 12th 23·50 | 50·29 | 60·50 | 36·84 | 73·00 | 50·00 | 67·46 | 55·56 | 61·51 | 11·90 | 18·00 | 6 |
| 7th, 30·50 | 48·27 | 59·00 | 29·67 | 70·00 | 45·00 | 65·76 | 52·22 | 58·99 | 13·51 | 20·00 | 4 |
| 2d, 18·66 | 39·40 | 54·67 | 23·34 | 65·00 | 30·00 | 53·90 | 44·64 | 49·27 | 9·25 | 16·00 | 2 |

*TABLE shewing the Mean of the Hygrometer and Thermometer—the Quantity of Rain which fell—the Number of Days on which Rain fell—and the Number of Days each Wind prevailed at Naples, during the Year* 1838.

| RAIN. | | HYGROMETER. | | | | | | THERMOMETER. | | | | | | | | No. of Days each Wind prevailed | | | | | | | |
|---|---|---|---|---|---|---|---|---|---|---|---|---|---|---|---|---|---|---|---|---|---|---|---|
| Number of Days on which Rain fell | Number of French Lines & Decimals of Rain during the Year. | Mean of the differences between the dry and wet Bulbs | Mean absolute Dryness according to Dr. Mason's calculation | Greatest absolute dryness observed during the year | Mean Dew Point | Highest Dew Point observed during a whole day | Lowest Dew Point observed—mean of a whole day. | Maximum of the Year | Minimum of the Year | Mean maximum | Mean minimum | Mean Temperature | Mean diurnal Variation | Greatest diurnal Variation | Least diurnal Variation | Days from South-east | Days from South | Days from South-west | Days from West | Days from North-west | Days from North | Days from North-east | Days from East |
| 137 | 505.21 | 4.96 | 11.54 | 37.33 | 48.22 | 65.67 | 23.34 | 83.00 | 30.00 | 66.01 | 54.35 | 60.18 | 11.56 | 24.00 | 2.00 | 43 | 57 | 103 | 49 | 29 | 25 | 43 | 16 |

These Tables were kept, if not with all the exactness I could wish, at least with as much regularity as my professional duties would permit—the observations were generally taken three times a day at the hours specified, or as near to these hours as I could conveniently do it. The following remarks may be useful, as explaining the manner in which the tables were kept.

I. The rain gauge, from which the quantity of rain is taken, was kept at the Royal Marine Observatory, about two miles from my house, and at an elevation of two hundred and sixty French feet above the level of the sea; the rainy days, however, were notes by myself each evening, and the crosses (×) in the column marked *prevailing winds,* shews what appeared to me to be the proportionate quantity of rain at the time it fell, from ×, a few drops, to × × × ×, heavy and long-continued rain. The measurement of the rain is made in French lines and decimals, and as I am not quite certain as to the proportion between French and English lines and inches, I have thought it best to give, in the table, the original measurement: during the year 1838, therefore, you will perceive that there were 137 days on which rain fell, and that the quantity of rain amounted in all to 505.21 lines French measurement: now if we reckon the French line as equal to 0.91 English, (which I believe to be pretty nearly the truth,) we shall then have 100:91::505.21:45.97 English inches, or within a fraction of 46 English inches. This quantity of rain is great in comparison with England; but the evaporation after showers is so rapid here, that the streets are generally quite dry in half an hour after the rain falls. I must also remark that the year 1838 was unusually wet, especially during the months of January, February, and March—and in the whole year of 1838 there fell nearly a fifth part more of rain than usually falls in one year—the average quantity of rain I believe to be about 36 inches.

II. The hygrometer I use is the same as that employed by the late Dr. Mason in making his observations in the Island of Madeira. It consists of two thermometers, the bulbs of which are covered with thin muslin or tafeta—the one bulb dry, the other kept constantly moistened with boiled or distilled water—the difference between these two thermometers noted three times a day, and the mean of the three observations taken, constitutes what is called the *mean of the differences observed,* in the first column under the head "Hygrometer." The second column under the head Hygrometer is intended to shew the degree of absolute dryness which exists in the air, which, according to Dr. Mason's calculation, is a fraction more than double, or as 7 to 3 to the difference observed. The third column, " the greatest absolute dryness," will shew what the greatest dryness has been during the day, and this is generally between the hours of 1 and 2 in the afternoon. The dew point is obtained by subtracting the mean absolute dryness in the second column from the mean temperature. Obs. I may here remark that the dampest time of the day is from sun-set to sun-rise, and that the maximum degree of saturation is usually about an hour before sun-rise. A little before sun-rise, however, the evaporation begins and goes on encreasing till 1 or 2 o'clock in the afternoon, at which time the dryness is usually at its greatest degree. About 4 o'clock in the afternoon the evaporation begins to diminish, and consequently the dampness to encrease, and this goes on till after sun-set, from which time till the morning the saturation of the air is at its greatest.

III. The thermometers I employ for obtaining the temperature are of two kinds—the temperature of the night, or the minimum of the 24 hours is taken with a spirit register thermometer, which is placed in a sheltered situation open to the North and East; but as it is impossible, from the position of my house, to keep the maximum register thermometer free from reflected heat at all hours of the day, I have preferred taking the maximum temperature by means of a common thermometer placed in the shade in a North-east aspect, between the hours of two and three in Summer, and between one and two in Winter, these being the hours of the day when the temperature is usually at its highest pitch. The thermometer which I employ for taking the temperature at nine, two, and ten o'clock, is the same which forms the dry bulb of the hygrometer. I use this thermometer not only because it saves time, but also because the bulb being covered with muslin, it is thereby better protected from reflected heat. Obs. It will be observed that the mean temperature for the year is given by me at 60.18, which is lower than that given by Sir James Clark, in his work on Climate, as the mean temperature of Naples. This circumstance is easily explained—my observations are made during the Summer months at *Sorrento* instead of Naples, and there the temperature is considerably lower than in the latter place, particularly during the months of July and August, at which time the country being covered with vines and vegetables, the temperature is not encreased as it is in Naples by terrestrial radiation.

IV. The peculiar position of Naples renders it very difficult if not impossible to state precisely the quarter from which the winds come: the town stands on the declivity of a hill, open to the sea on the South, but defended on the side towards the land, not only by the hill on which it stands, but also by higher mountains behind this, which have a considerable influence in altering the direction of the wind, so that often while, in one part of the town, the wind appears to be from the North, in another part it will be from the North-east, and in a third from the North-west. Although, therefore, I have, in these tables, divided the winds into eight points, still, as affecting the climate, and thereby producing an influence on the health of the inhabitants, it will be sufficient for our purpose to class them under two different heads; the first comprehending the land-winds, and the other the sea-winds. Under the name of land-winds I include all winds from the North of West round to the East; and under the opposite denomination of sea-winds I include all from the South-east, South, and South-west. The West wind ought properly to form a division by itself as being different from both the others—neither so cold and exciting as the land-winds, nor so wet and stormy as the wind from the sea; this wind, however, is unfortunately of so short continuance, that it can hardly be considered as exerting any influence on the salubrity or insalubrity of Naples. The first class of winds, then, the land-winds, are all those which come from the North-west, North, North-east, and East. They are most frequent during the vernal and autumnal months, and this year they were most prevalent during the months of December, November, October, March, February, and January, in the order I have now stated them: these winds are dry and cold, irritating to the lungs of patients in the first stage of tubercular phthisis, and injurious to many patients who suffer from spasmodic asthma, rheumatism, or from irritation of the mucous membranes of the stomach, intestines, and air-passages. On the other hand, to patients who suffer from simple debility, without any organic affection, and to those who are affected with particular kinds of nervous headaches, and to whom the air of England and even that of Rome feels heavy and oppressive—to patients with relaxation of the muscular fibre brought on by uterine or other discharges—in fact, to all patients who require a bracing air, or whose complaints are benefitted by tonic medicines—to all such this land-wind is most agreeable and beneficial, and it is surprising how suddenly persons, who can hardly stir from the sofa during the prevalence of the sea-wind, regain their strength and spirits as soon as it comes round to the land. It is, however, a dangerous wind to patients who suffer from pulmonary disease or from organic disease in general, more especially as the variations of temperature are always greatest while this wind prevails, the difference of temperature between the day and night, or, in other words, the diurnal variation, being then at its maximum, as well as the difference of temperature between the sun and shade; for as the sky is usually at this time very clear without any clouds to intercept the rays of the sun, its power is usually great, while the wind coming from the snowy Appenines renders the shade unusually cold.

The sea-winds are by far the most prevalent, and of these the South-west is the most frequent. In 1838 the sea-winds were in proportion to the land-winds, as 250 to 115. In Summer the sea-breeze is from the South-west, and usually blows from 10, a. m., till 5, p. m.; and in Winter it blows more than two-thirds of the time. In Winter the rain and strongest winds usually come from the South-west. During the continuance of these winds the mean temperature is greater, and the diurnal variations less, than when the opposite winds prevail; they are therefore considered as being well suited to patients with pulmonary diseases, rheumatism, and chronic bronchitis; but, on the whole, the climate of Naples is too variable for pulmonary diseases.

---

### MR. STILON (OF MALTA) ON THE BLIND CATHETER.

Every medical man who has been engaged for any time in the practice of his profession, must be fully aware how indispensable for the proper and conscientious discharge of his duties is a real zeal and anxious interest in the welfare of his patients. How many inventions, how many solid improvements in science may be traced up to this cause, and whatever merit the new form of catheter, represented in the accompanying plate, may have, it owes its origin to this cause, having suggested itself to me during an anxious attendance on a case of a very trying nature.

In the month of October of the year 1838, I was requested to attend Mr. Gilio of this city, a person in easy circumstances, and of about 80 years of age. I found him labouring under severe retention of urine, and in great pain. He referred his complaint to piles, from which he had been suffering many years, and which frequently appearing externally, brought on copious hæmorrhage, and occasioned him great distress.

For some time before I was called he had observed that he voided but a small quantity of urine, and attributing this to the pressure of the hæmorrhoidal tumor, had had leeches applied. The relief which followed was only trifling, and in no way proportioned to his expectations, as on any former occurrence of the same symptoms the application of a few leeches had always proved most effectual.

The urine, in the mean time, kept accumulating; the bladder was soon greatly distended above the pubis, and the unfortunate patient was incessantly tormented with a desire to micturate.

The case was sufficiently urgent, and I immediately introduced a gum elastic catheter, which brought away a great quantity of urine mixed with blood. In introducing the instrument I met with some difficulty at the neck of the bladder, and concluded that that was the seat of the obstruction. On examining the instrument, after having withdrawn it, I found the eyes, or apertures, at its rounded extremity, filled with coagulated blood; however there was every reason for supposing that the bladder had been completely emptied.

For several days I was obliged to introduce the catheter twice a day. But on introducing it one morning I was surprised by not seeing any urine flow from it. I took out the wire, still no urine issued—withdrawing the catheter, I found its apertures completely closed with coagula, which obstinately appeared to oppose the passage of the urine. I now hoped, by re-introducing the catheter several times, and freely moving it about, I might succeed in breaking up the coagula. I tried, but in vain; and as the case was becoming very urgent, I desired a consultation should take place. This was immediately attended to, and while the other medical men were present, I introduced a longer catheter than the one I had used before, and so succeeded in extracting a moderate quantity of urine mixed with blood. On account of the former difficulties it was thought advisable by the other gentlemen present, as well as myself, to leave the catheter in during the night. As the catheter did not act at all, I withdrew it at my visit next morning, and found it filled with coagulated blood. After cleaning it well I again introduced it, but to no purpose, for not a single drop of urine followed. The symptoms were now so urgent, that there was every reason to fear for the patient's life. Animated however by the well-known saying,—"Nihil tam arduum quod non expugnat pertinax operosa ac diligens cura," I did not lose all hope. The symptoms seemed to me clearly to indicate the nature of the obstacle. There were evidently coagula either in the cavity of the bladder, or in the canal of the urethra; and this by closing the apertures of the catheter, of course prevented the flow of urine through it. After pondering for some time on the peculiar circumstances of the case, it struck me that if I could modify the catheter, so as to have in it a portion that might push aside the coagula, while another gave passage to the urine, it would answer the purpose, and enable me to give immediate relief. The printed form of the instrument, which I inclose with this, soon suggested itself to my mind, and I hastened at once to have one made after my direction. I shall not attempt to describe the anxiety with which I again visited my patient; the symptoms were more urgent than ever, and he was crying aloud for relief. I introduced the modified catheter, and, to my great satisfaction, the urine immediately flowed from it in great quantity and with the greatest ease. Every successive trial of it on the following days met with the same success, and further reflections and experience has fully convinced me of the advantages which such a form of catheter will be found to present—the more it is generally known, and the more it is applied to the many cases of a similar nature which occur in the course of medical practice. This conviction induces me to beg of you to publish the accompanying design, as well as the history of the case, which suggested the modification, and should feel highly gratified if it in any way meet the general approbation of the profession.

I shall conclude by some few remarks on the instrument itself. It can be introduced without the least fear of limiting the mucous membrane of the urethra or bladder. The screw which runs through it perfectly insures this, and neither during its introduction, nor during its extraction, can it present the least roughness. Should it be necessary to leave it in the bladder for any length of time, its internal surface will remain perfectly clean so long as its extremity is kept closed (as it ought to be), and only opened when the bladder is to be relieved from its contents. The perfect smoothness and firm support of its extremity, the mechanism of the screw, and the nice adaptation of its different parts, seem to me to render it a far superior and far preferable instrument to those of Petit and Franco, which may be said to have some resemblance to mine.

